# Efficient Time-Domain Imaging Processing for One-Stationary Bistatic Forward-Looking SAR Including Motion Errors

**DOI:** 10.3390/s16111907

**Published:** 2016-11-12

**Authors:** Hongtu Xie, Shaoying Shi, Hui Xiao, Chao Xie, Feng Wang, Qunle Fang

**Affiliations:** 1 Department of Air/Space-based Early Warning Equipment, Air Force Early Warning, Wuhan 430019, China; Shisy2006@163.com (S.S.); callmexh@163.com (H.X.); superxpapa@126.com (C.X.); qiulei2013@nudt.edu.cn (F.W.); phylqfang@126.com (Q.F.); 2 College of Electronic Science and Engineering, National University of Defense Technology, Changsha 410073, China

**Keywords:** efficient time-domain algorithm (ETDA), one-stationary bistatic forward-looking synthetic aperture radar (OS-BFSAR), imaging processing, motion errors, polar grids

## Abstract

With the rapid development of the one-stationary bistatic forward-looking synthetic aperture radar (OS-BFSAR) technology, the huge amount of the remote sensing data presents challenges for real-time imaging processing. In this paper, an efficient time-domain algorithm (ETDA) considering the motion errors for the OS-BFSAR imaging processing, is presented. This method can not only precisely handle the large spatial variances, serious range-azimuth coupling and motion errors, but can also greatly improve the imaging efficiency compared with the direct time-domain algorithm (DTDA). Besides, it represents the subimages on polar grids in the ground plane instead of the slant-range plane, and derives the sampling requirements considering motion errors for the polar grids to offer a near-optimum tradeoff between the imaging precision and efficiency. First, OS-BFSAR imaging geometry is built, and the DTDA for the OS-BFSAR imaging is provided. Second, the polar grids of subimages are defined, and the subaperture imaging in the ETDA is derived. The sampling requirements for polar grids are derived from the point of view of the bandwidth. Finally, the implementation and computational load of the proposed ETDA are analyzed. Experimental results based on simulated and measured data validate that the proposed ETDA outperforms the DTDA in terms of the efficiency improvement.

## 1. Introduction

Nowadays, the synthetic aperture radar (SAR) has a major advantage for the high resolution imaging processing in all time and all weather conditions, and plays a very significant role in remote sensing, geosciences, surveillance and reconnaissance applications, and thus it is widely investigated in both civilian and military fields [1,2,3,4,5,6].

Bistatic forward-looking SAR (BFSAR) [7] is a special bistatic SAR (BSAR) system [8,9], where the radar system works in the forward-looking mode compared with the traditional BSAR system working in the side-looking mode. It not only inherits the advantages of the BSAR system, such as reduced vulnerability for military applications, exploiting the additional information and improving the detectability of the stealth targets, but also carries out high resolution scene imaging in the forward direction [10], and therefore it has gained wide attention in missile navigation, war-field reconnaissance, and forward-looking imaging. Recently, several countries have carried out the experiments on BFSAR imaging processing, and some excellent results were obtained, such as the BFSAR experiment with a fixed ground-based receiver working in the forward-looking mode [11,12], the BFSAR experiment with a vehicle-based transmitter and a receiver on board an ultra-light aircraft [13,14,15], and the BFSAR experiment using the TerraSAR-X as the transmitter and the airborne phased array multifunctional imaging radar (PAMIR) as the receiver [16,17]. One-stationary BFSAR (OS-BFSAR) is regarded as a SAR system combining the one-stationary BSAR (OS-BSAR) and BFSAR systems, which has a stationary radar (transmitter or receiver) fixed on the top of a high tower or mountain and a moving radar (receiver or transmitter) placed at a vehicle/airborne/spaceborne platform. The OS-BFSAR system not only inherits the advantages of the OS-BSAR and BFSAR systems, but also is subject to the difficulties of the imaging processing due to its special configuration, i.e., the huge amount of echo data, large spatial-variance, serious range-azimuth coupling and complicated motion errors. These make the precise imaging processing of the OS-BFSAR data more complicated.

Like monostatic SAR imaging algorithms, BSAR imaging algorithms can be also categorized into two groups: frequency-domain algorithms (FDAs) and time-domain algorithms (TDAs). The FDAs generally aim to minimize the time of the imaging processing. However, this aim can induce many limitations such as the bandwidth, integration time, motion errors, assumptions and approximations in the imaging processing, real-time imaging processing, memory requirements and so on, which may restrict the application of the FDAs. Thus, FDAs are only available for some particular SAR imaging processing cases. Recently, monostatic FDAs [1,2,3,4], which include the range Doppler algorithm (RDA) [18,19,20], the Omega-k algorithm (OKA) [21,22,23,24], the chirp scaling algorithm (CSA) [25,26,27] and nonlinear CSA (NLCSA) [28,29,30,31], have been extended for BSAR imaging processing. Among the above-mentioned methods, RDA, OKA and CSA are only available for the azimuth-invariant BSAR imaging processing, thus they cannot always satisfy the precise imaging processing for the all BSAR configurations in practice, especially for azimuth-variant BSAR systems. NLCSA and its modifications have been used to implement imaging processing for the different BSAR configurations, but there are some approximations of handling the spatial-variance, range-azimuth coupling and motion errors, which may cause large phase errors in some particular BSAR imaging processing cases. Thus, the FDAs are only valid for a limited number of BSAR systems, which don’t satisfy the precise imaging processing for the OS-BFSAR due to the large spatial variances, serious range-azimuth coupling and complicated motion errors. For the OS-BFSAR, if the minimum phase error and the highest resolution are required, processing such BSAR data must rely on TDAs, which can avoid the limitations of the FDAs.

The TDAs include the direct TDA (DTDA), like the backprojection algorithm (BPA) [32], and the efficient TDA (ETDA), like the fast BPA (FBPA) [33,34,35] and fast factorized BPA (FFBPA) [36]. DTDA [32] is considered a linear transformation to reconstruct the SAR scene from the radar echoes, and thus it can be applied directly to monostatic and bistatic imaging processing with perfect focusing performance. Importantly, it can precisely accommodate the abovementioned problems in the OS-BFSAR imaging processing. Moreover, it can offer the further advantage of the precise disposal of the irregular sampling, particularly useful in the OS-BFSAR system. However, the DTDA has a higher computational load, which may prevent its use as a standard method for monostatic and bistatic imaging processing. To reduce the higher computational load, the efficient implementation of the TDA has been applied for the monostatic SAR imaging processing (i.e., FBPA [33]), which is based on a two-step split of the synthetic aperture. The FBPA method, including the derivation of Nyquist requirements for the linear track SAR imaging processing was presented in [34]. A quad-tree-based FBPA for the arbitrary motion SAR imaging processing was proposed in [35], offering the idea of splitting the imaging processing into multiple stages. All the developments of the monostatic FBPA converged into the FFBPA [36], which is an optimum method benefiting from the multiple stage factorizations working in an efficient geometry in terms of the image sampling. These ETDAs are based on the subaperture processing techniques, which can keep all the advantages of the DTDA but with a reduced computational load. With the rapid development of the BSAR technologies in recent years, the monostatic ETDAs have been extended to the BSAR imaging processing, which are classified into two kinds: the bistatic FBPA [37,38,39,40,41,42] and bistatic FFBPA [43,44,45,46,47,48]. A study on the ability of the bistatic FBPA to handle the bistatic range history precisely was developed in [37]. In [38], a bistatic FBPA based on the subaperture and subimages has been presented for BSAR imaging processing, which required an intermediate processing step involving beamforming from the radar echoes. The phase errors caused by the approximations in this bistatic FBPA was analyzed in [39] to provide a good trade-off between the phase error and the computational load in imaging processing.

Another bistatic FBPA based on the subaperture and polar grid was proposed in [40], which required mapping the radar echoes into polar grids instead of the beamforming from the radar echoes as an intermediate processing step. In this research, the reconstruction of the SAR scene was recommended in a ground plane instead of in a slant-range plane, and the polar range coordinate and polar angular coordinate necessarily depended on both transmitter and receiver tracks, but the sampling requirements for polar grids were derived in [41] for the linear trajectory BSAR system. The bistatic FBPA for the OS-BSAR imaging processing was proposed in [42], which also represented the subimages in the Cartesian ground plane. However, the oversampling ratios of the subimages in the azimuth and range directions were uncertain, thus the sampling requirements for the subimages weren’t optimal. The bistatic FFBPA was first used to the OS-BSAR imaging processing in [43], but only the experimental results were given. Application of the bistatic FFBPA to process the BSAR data was presented in [44], which gave the basic principles of the bistatic FFBPA, whereas the details of its implementation weren’t given.

Another bistatic FFBPA has been applied for a space-borne-airborne BSAR case [45], and it first represented the subimages in the elliptical polar grids to reduce the computational load. However, the sampling requirement for the elliptical polar grids was derived not only for the linear track BSAR system, but also for the preferred BSAR case of the radars with the higher angular velocity. Considering the motion errors, the bistatic FFBPA with the derivation of the sampling requirement for the elliptical polar grids was developed in [46,47]. However, for the bistatic FFBPA given in [45,46,47], the elliptical polar range coordinate and polar angular coordinate were only referenced to the transmitter or receiver track, thus the applicability of these sampling requirements was limited in some instances. The authors of [38] presented a bistatic FFBPA for the linear trajectory BSAR imaging processing in [48], which gave the requirements for splitting the subaperture and subimage, while the sampling requirements of the beams for the corresponding subimages were not given. In [49], the bistatic FBPA and FFBPA based on the subapertures and local polar coordinates were proposed for the general bistatic airborne SAR systems, which in fact was a synthesis of the research given in [40] and [41]. Similarly, the motion errors was not considered in the derivation of the sampling requirements for polar grids, which cannot offer a near-optimum tradeoff between the imaging precision and efficiency in the BSAR imaging processing in practical. It is known that the bistatic ETDA in [37,38,39,40,41,42,43,44,45,46,47,48,49] were developed for the traditional bistatic side-looking SAR (BSSAR) imaging processing. However, to our knowledge, the ETDA for the OS-BFSAR imaging processing has hardly been investigated in earlier publications, thus it may still be desirable for practical OS-BFSAR data processing.

Based on these previous works, this paper explores an ETDA considering motion errors for the OS-BFSAR imaging processing based on the subaperture and polar grid processing. This method represents the subimages on polar grids in the ground plane instead of the slant-range plane, and it is referenced to the positions of both moving and stationary radars. It can not only accurately accommodate the large spatial variances, serious range-azimuth coupling and motion errors, but also improve greatly the imaging efficiency with respect to the DTDA. First, the OS-BFSAR imaging geometry is built, and the DTDA for the OS-BFSAR imaging processing is provided, which lays the foundation for the proposed ETDA. Second, the polar grids of the subimages are defined, and then the subaperture imaging processing in the ETDA is derived. The sampling requirements considering motion errors for the polar grids are derived from the point of view of the bandwidth, which can offer a near-optimum tradeoff between the imaging precision and efficiency. Third, the implementation and computational load of the proposed ETDA are analyzed, and then the speed-up factor of the proposed ETDA with respect to the DTDA is derived. Finally, the presented ETDA is tested and validated by experimental results based on the simulated and measured OS-BFSAR data.

This paper is organized as follows: Section 2 reviews the bistatic DTDA for the OS-BFSAR imaging processing. The details of the proposed ETDA, including the definition of the polar grids, subaperture imaging processing, sampling requirements for the polar grids, implementation and computational load, are presented in Section 3. Experimental results and the corresponding analysis based on simulated and measured data are given in Section 4. Conclusions are drawn in Section 5.

## 2. DTDA for OS-BFSAR Imaging Processing

### 2.1. Imaging Geometry

**Figure 1:**
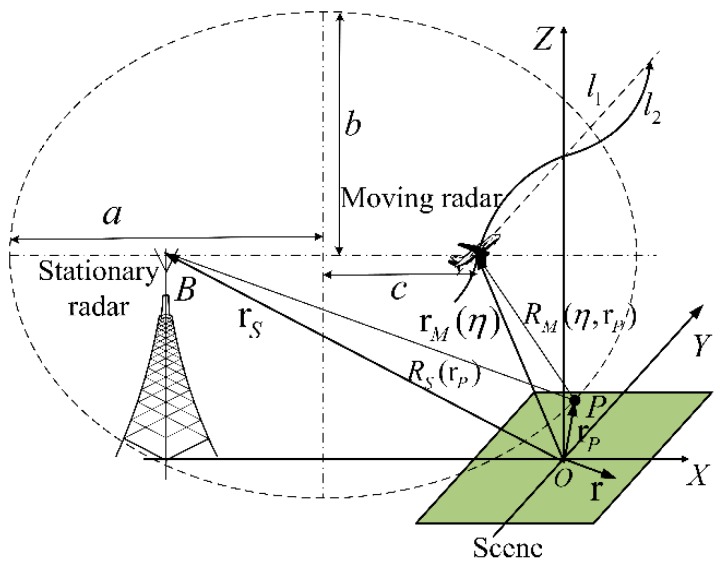
Imaging geometry of the OS-BFSAR system including the motion errors.

The imaging geometry of the OS-BFSAR system including the motion errors is shown in Figure 1. The straight line *l*_1_ is the ideal track of the moving radar, and its actual track is the curve *l*_2_. The position of the moving radar is **r***_M_*(*η*) = (*x_M_*(*η*),*y_M_*(*η*),*z_M_*(*η*)) at the slow time(azimuth time) *η*, while the position of the stationary radar is **r***_S_* = (*x_S_*,0,*z_S_*). Suppose that the illuminating beam of the moving radar is always covered by that of the stationary radar in order to insure the synchronization of this OS-BFSAR system, and the moving radar operates in the forward-looking spotlight mode. *P* is assumed to be an arbitrary scattering target in the scene, and its position is **r***_P_*. The distances from the moving and stationary radars to the scattering target *P* at the slow time *η* are *R_M_*(*η*,**r***_P_*) and *R_S_*(**r***_P_*), respectively. Therefore, the bistatic distance from the scattering target *P* to the moving and stationary radar at the slow time *η* is:

(1) $$R(\eta ,r_{P})=R_{M}(\eta ,r_{P})+R_{S}(r_{P})=\left|r_{P}-r_{M}(\eta )\right|+\left|r_{P}-r_{S}\right|$$

Provided that the transmitted signal is *p*(*τ*), and then the received signal of the target *P* is:

(2) $$s(\tau ,\eta )=\sigma_{P}\cdot p\left[\tau -R(\eta ,r_{P})/c_{0}\right]$$

where, *τ* is the fast time, *σ_P_* is the scattering coefficient of the scattering target *P*, and *c*_0_ is the speed of light. Therefore, the range-compressed signal of the scattering target *P* is:

(3) $$s_{rc}(\tau ,\eta )=\sigma_{p}\cdot p_{rc}\left[B\left(\tau -R(\eta ,r_{P})/c_{0}\right)\right]$$

where *p_rc_*[·] is the range-compressed pulse, and *B* is the transmitted signal bandwidth.

### 2.2. DTDA for OS-BFSAR Imaging

The DTDA (i.e., the BPA) can be considered as a direct transformation process from the radar echoes into a complex SAR image, and therefore it can be applied directly for the OS-BFSAR imaging processing without any modification. Unlike the monostatic DTDA, the backprojection (BP) of the radar echoes in the bistatic DTDA for the OS-BFSAR imaging processing is carried out over an ellipsoidal basis. In Figure 1, *a* and *b* are the major and minor axes of the dashed ellipse, whose foci are determined by the positions of the considered moving and stationary radars. Based on the major and minor axes (*a* and *b*), the linear eccentricity can be defined as $c=\sqrt{a^{2}+b^{2}}$. **r** = (*x,y*,0) is assumed to be an arbitrary sample in the scene, and then the value of the SAR image at the sample **r** calculated by the bistatic DTDA is given by [32]:

(4) $$\begin{array}{cc} I(r) & =\int_{\eta_{c}-T/2}^{\eta_{c}+T/2}s_{rc}\left(R(\eta ,r)/c_{0},\eta \right)\cdot \exp\left[j2\pi fR(\eta ,r)/c_{0}\right]d\eta \\ & =\int_{\eta_{c}-T/2}^{\eta_{c}+T/2}\sigma_{P}\cdot p_{rc}\left[B\left(\left(R(\eta ,r)-R(\eta ,r_{P})\right)/c_{0}\right)\right]\cdot \exp\left[j2\pi fR(\eta ,r)/c_{0}\right]d\eta \end{array}$$

where *η_c_* is the synthetic aperture center time of the moving radar, *f* is the radar frequency, and *T* is the integration time.

## 3. ETDA for OS-BFSAR Imaging Processing

To reduce the computational load of the bistatic DTDA, the efficient implementation of the bistatic DTDA (i.e., ETDA) for OS-BFSAR imaging processing is presented. The proposed ETDA is developed from the research described in [40,41] based on the subaperture and polar grid processing, but the motion errors is considered in the derivation of the sampling requirements for polar grids in the proposed ETDA, which can offer a near-optimum tradeoff between the imaging precision and efficiency. Similar to the ETDA in [40,41], the polar grids in the ground plane instead of the slant-range plane are highly recommended for calculating the subimages in the proposed ETDA, since there is no exact slant-range plane for the BSAR configurations. Moreover, the polar grids of the subimages and the SAR image grids in the same plane can also simplify the calculation of the travel distance of a radar pulse from the transmitter and receiver to the scene.

Similar to the ETDA for the BSSAR imaging processing in [46,47], the proposed ETDA is able to accommodate the non-ideal track of the moving radar (i.e., compensating the motion error of the moving radar), but does not increase the computational load. In the first processing stage, the motion error can be accurately compensated in the subaperture imaging using the bistatic ETDA. In other words, the motion error is corrected for each BP data line by computing the bistatic range from each subaperture position of the moving radar via the polar grid of subimages to the position of the stationary radar. In the successive processing stage, the higher resolution subimage is interpolated from the lower resolution subimages in the previous stage. Therefore, the finer motion error compensation is included by the proposed ETDA as the resolution successively improves through the processing stages [46,47].

### 3.1. DTDA with Subaperture and Polar Grid Processing

**Figure 2:**
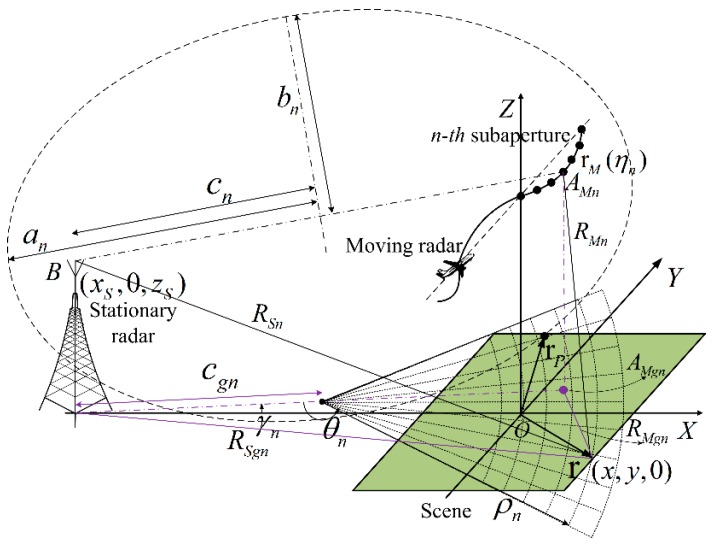
DTDA with the subperture and polar grid processing.

The OS-BFSAR imaging geometry for the bistaticDTDA with the subaperture and polar grid processing is shown in Figure 2. For the *n*-th subaperture of the moving radar, *A_Mn_* is the *n*-th subaperture center of the moving radar at the *n*-th subaperture center time *η_n_*. The position vector of the moving radar at the slow time *η_n_* is **r***_M_*(*η_n_*) = (*x_M_*(*η_n_*),*y_M_*(*η_n_*),*z_M_*(*η_n_*)), and its projection in the X-Y plane is *A_Mgn_* with the position vector **r***_Mg_*(*η_n_*) = (*x_M_*(*η_n_*),*y_M_*(*η_n_*),0). The distances from the moving and stationary radars to the sample **r** at slow time *η_n_* are *R_Mn_* and *R_Sn_*, and its projection in the X-Y plane are *R_Mgn_* and *R_Sgn_*, respectively. *a_n_*, *b_n_* and *c_n_* have the similar physical meanings as *a*, *b* and *c* in Figure 1, respectively, and the projection of the linear eccentricity *c_n_* is *c_gn_*. Similar to the monostatic DTDA, the polar coordinates (*ρ_n_*,*θ_n_*) of the sample **r** are defined in the ground plane as following [41]. First, the origin of the polar grid is defined as the projection of the center point of the link line between the stationary radar position *B* and considered moving radar subaperture center position *A_Mn_*. Second, the polar range *ρ_n_* is defined as the distance between the origin of the polar grid and the sample **r**, and the polar angle *θ_n_* is defined as the angle from the linear eccentricity projection *c_gn_* to the polar range *ρ_n_*. Thus, the polar coordinates (*ρ_n_*,*θ_n_*) of the sample **r** are determined by:

(5) $$\left\{ \begin{array}{l} \rho_{n}=\sqrt{{\left[\frac{x_{S}+x_{M}(\eta_{n})}{2}-x\right]}^{2}+{\left[\frac{y_{M}(\eta_{n})}{2}-y\right]}^{2}}\\ \theta_{n}=\arccos\left(\frac{\rho_{n}^{2}+c_{gn}^{2}-({\left(x_{S}-x\right)}^{2}+y^{2})}{2\rho_{n}c_{gn}}\right),\;\theta_{n}\in [0,\pi ] \end{array}\right.$$

It is well known that the angle *θ_n_* in Equation (5) is calculated based on the law of cosine. Using the above equation, we can get the Cartesian coordinates of the sample **r**, which are given by:

(6) $$\left\{ \begin{array}{l} x=\left(x_{S}+x_{M}(\eta_{n})\right)/2+\rho_{n}\cos(\pi \pm (\gamma_{n}+\theta_{n}))\\ y=y_{M}(\eta_{n})/2+\rho_{n}\sin(\pi \pm (\gamma_{n}+\theta_{n})) \end{array}\right.\;where\;\left\{ \begin{array}{l} +\;when\;y_{M}(\eta_{n})\geq 0\\ -\;when\;y_{M}(\eta_{n})<0 \end{array}\right.$$

It can be found that the sign ± in Equation (6) depends on the relative positions between the subaperture center *A_Mn_* and the sample **r** in the polar subimage. Here, *γ_n_* is the angle from the linear eccentricity projection *c_gn_* to the X axis, which can be given by:

(7) $$\gamma_{n}=\arccos\left(\frac{x_{S}^{2}+4c_{gn}^{2}-{\left(x_{M}(\eta_{n})\right)}^{2}-{\left(y_{M}(\eta_{n})\right)}^{2}}{-4x_{S}c_{gn}}\right)$$

Similarly, the polar coordinates (*ρ_np_,θ_np_*) of the scattering target *P* can be defined. Let *R*(*η*,*ρ_np_,θ_np_*) = *R*(*η*,**r***_p_*) and *R*(*η*,*ρ_n_,θ_n_*)= *R*(*η*,**r**), the value of the polar subimage at the sample (*ρ_n_,θ_n_*) for the *n*-th subaperture imaging processing is calculated as:

(8) $$\begin{array}{cc} I_{n}(\rho_{n},\theta_{n}) & =\int_{\eta_{n}-T_{n}/2}^{\eta_{n}+T_{n}/2}s_{rc}\left[\frac{R(\eta ,\rho_{n},\theta_{n})}{c_{0}},\eta \right]\cdot \exp\left[\frac{j2\pi fR(\eta ,\rho_{n},\theta_{n})}{c_{0}}\right]d\eta \\ & =\int_{\eta_{n}-T_{n}/2}^{\eta_{n}+T_{n}/2}\sigma_{P}p_{rc}\left[\frac{B\left(R(\eta ,\rho_{n},\theta_{n})-R(\eta ,\rho_{np},\theta_{np})\right)}{c_{0}}\right]\cdot \exp\left[\frac{j2\pi fR(\eta ,\rho_{n},\theta_{n})}{c_{0}}\right]d\eta \end{array}$$

where *T_n_* is the integration time of the *n*-th subaperture.

### 3.2. Sampling Requirements for Polar Grids Considering Motion Errors

**Figure 3:**
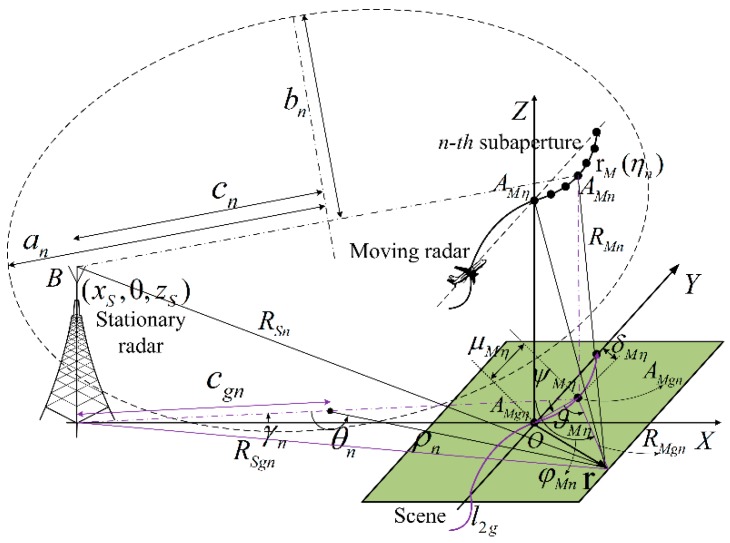
Bistatic range calculation in the proposed ETDA.

From the point of view of the bandwidth, the sampling requirements for the polar grids can be derived by calculating the bistatic range from the moving and stationary radars to the sample (*ρ_n_*,*θ_n_*). The bistaitc range calculation of the sample (*ρ_n_*,*θ_n_*) in the *n*-th subaperture imaging processing is now shown in Figure 3. *A_Mη_* is the moving radar position at the slow time *η*, and the *n*-th subaperture integration time is *η*∈ (*η_n_* – *T_n_*/2, *η_n_* + *T_n_*/2). *l*_2*g*_ is the projection of the real flight track *l*_2_ of the moving radar in the X-Y plane, and *A_Mgη_* is the projection of the moving radar position *A_Mη_* in the X-Y plane. *μ_Mη_* is the distance between the moving radar position projections *A_Mgn_* and *A_Mgη_* along the Y axis direction, and *δ_Mη_* is the distance between the moving radar position projections *A_Mgn_* and *A_Mgη_* along the X axis direction, and therefore the length of the straight line *A_Mgn_A_Mgη_* is $d_{M\eta }=\sqrt{\mu_{M\eta }^{2}+\delta_{M\eta }^{2}}$. *ϑ_Mη_* is the angle between straight line *A_Mgn_A_Mgη_* and the range projection *R_Mgn_*, and *ψ_Mη_* is the angle between the straight line *A_Mgn_A_Mgη_* and the linear eccentricity projection *c_gn_*. *ϕ_Mn_* is defined as the angle between the range projection *R_Mgn_* and the linear eccentricity projection *c_gn_*.

From the imaging geometry in Figure 3, the bistatic distance from the moving and stationary radars to the sample (*ρ_n_*,*θ_n_*) at the slow time *η* can be computed and expanded using the Taylor series, which is given by:

(9) $$\begin{array}{cc} R(\eta ,\rho_{n},\theta_{n}) & =R_{M}(\eta ,\rho_{n},\theta_{n})+R_{S}(\rho_{n},\theta_{n})\\ & =\sqrt{R_{Mgn}^{2}+d_{M\eta }^{2}-2R_{Mgn}d_{M\eta }\cos(\vartheta_{M\eta })+z_{M}^{2}(\eta )}+\sqrt{R_{Sgn}^{2}+z_{S}^{2}}\\ & =R_{Mgn}+\;R_{Sgn}-d_{M\eta }\cos(\vartheta_{M\eta })+\frac{d_{M\eta }^{2}\sin^{2}(\vartheta_{M\eta })}{2R_{Mgn}}+\frac{z_{M}^{2}(\eta )}{2R_{Mgn}}+\frac{z_{S}^{2}}{2R_{Sgn}}+\cdot \cdot \cdot \end{array}$$

where, *R_M_*(*η*,*ρ_n_,θ_n_*) is the distance from the moving radar position *A_Mη_* to the sample (*ρ_n_,θ_n_*), and *R_S_*(*ρ_n_,θ_n_*) is the distance from the stationary radar position to the sample (*ρ_n_,θ_n_*).

For the far-field OS-BFSAR imaging, it is reasonable to assume that the range *d_Mη_* and height *z_M_*(*η*) are much smaller than the range *R_Mgn_*, and the height *z_S_* is much smaller than the range *R_Sgn_*. Therefore, all the terms, except the first three terms of Equation (9) are approximated to zeros. Taking only the first three terms of Equation (9) into account, Equation (9) can be approximated as:

(10) $$\begin{array}{cc} R(\eta ,\rho_{n},\theta_{n}) & \approx R_{Mgn}+\;R_{Sgn}-d_{M\eta }\cos(\vartheta_{M\eta })\\ & \approx \sqrt{\rho_{n}^{2}+c_{gn}^{2}+2\rho_{n}c_{gn}\cos(\theta_{n})}+\sqrt{\rho_{n}^{2}+c_{gn}^{2}-2\rho_{n}c_{gn}\cos(\theta_{n})}\\ & \;-d_{M\eta }\cos(\vartheta_{M\eta }) \end{array}$$

According to [41], it can be clearly found that *R_Mgn_* + *R_Sgn_*, is nearly a constant with respect to the polar angle *θ_n_* but not to the polar range *ρ_n_* for the *n*-th subaperture imaging processing. From the imaging geometry in Figure 3, cos(*ϑ_Mη_*) in Equation (10) can then be calculated as follows:

(11) $$\begin{array}{cc} \cos(\vartheta_{M\eta }) & =\cos(\varphi_{Mn}-\psi_{M\eta })\\ & =\cos(\varphi_{Mn})\cos(\psi_{M\eta })+\sin(\varphi_{Mn})\sin(\psi_{M\eta })\\ & =\frac{R_{Mgn}^{2}+c_{gn}^{2}-\rho_{n}^{2}}{2R_{Mgn}c_{gn}}\cos(\psi_{M\eta })+\frac{\rho_{n}\sin(\theta_{n})}{R_{Mgn}}\sin(\psi_{M\eta })\\ & =\frac{\left(c_{gn}+\rho_{n}\cos(\theta_{n}))\cos(\psi_{M\eta }\right)}{\sqrt{\rho_{n}^{2}+c_{gn}^{2}+2\rho_{n}c_{gn}\cos(\theta_{n})}}+\frac{\rho_{n}\sin(\theta_{n})\sin(\psi_{M\eta })}{\sqrt{\rho_{n}^{2}+c_{gn}^{2}+2\rho_{n}c_{gn}\cos(\theta_{n})}}\\ & =\frac{c_{gn}\cos(\psi_{M\eta })+\rho_{n}\cos(\theta_{n}-\psi_{M\eta })}{\sqrt{\rho_{n}^{2}+c_{gn}^{2}+2\rho_{n}c_{gn}\cos(\theta_{n})}} \end{array}$$

Substituting Equation (11) into Equation (10), the expression in Equation (10) can be written as:

(12) $$\begin{array}{cc} R(\eta ,\rho_{n},\theta_{n}) & \approx \sqrt{\rho_{n}^{2}+c_{gn}^{2}+2\rho_{n}c_{gn}\cos(\theta_{n})}+\sqrt{\rho_{n}^{2}+c_{gn}^{2}-2\rho_{n}c_{gn}\cos(\theta_{n})}\\ & \;-d_{M\eta }\frac{c_{gn}\cos(\psi_{M\eta })+\rho_{n}\cos(\theta_{n}-\psi_{M\eta })}{\sqrt{\rho_{n}^{2}+c_{gn}^{2}+2\rho_{n}c_{gn}\cos(\theta_{n})}} \end{array}$$

The two-dimensional Fourier transforms of the subimage *I_n_*(*ρ_n_*,*θ_n_*) in Equation (8) with respect to the polar range *ρ_n_* and polar angle *θ_n_* is given by:

(13) $$I_{FTn}(k_{\rho_{n}},k_{\theta_{n}})=\iint I_{n}(\rho_{n},\theta_{n})\exp\left[-j2\pi (k_{\rho_{n}}\rho_{n}+k_{\theta_{n}}\theta_{n})\right]d\rho_{n}d\theta_{n}$$

where $k_{\rho_{n}}$ and $k_{\theta_{n}}$ are the wavenumbers corresponding to the polar range *ρ_n_* and polar angle *θ_n_*, respectively. According to [34], the Fourier transform can be computed accurately using the stationary phase principle. Substituting Equation (8) into Equation (13), and then the stationary phase condition is given by:

(14) $$\left\{ \begin{array}{l} \frac{\partial \left(2\pi fR(\phi ,\rho_{n},\theta_{n})/c_{0}-2\pi k_{\rho_{n}}\rho_{n}\right)}{\partial \rho_{n}}=0\\ \frac{\partial \left(2\pi fR(\phi ,\rho_{n},\theta_{n})/c_{0}-2\pi k_{\theta_{n}}\theta_{n}\right)}{\partial \theta_{n}}=0 \end{array}\right.$$

From the Equations (10), (12) and (14), the wavenumber $k_{\rho_{n}}$ can be calculated by:

(15) $$\begin{array}{cc} k_{\rho_{n}} & =\frac{f}{c_{0}}\frac{\partial \left(R(\eta ,\rho_{n},\theta_{n})\right)}{\partial \rho_{n}}\\ & =\frac{f}{c_{0}}[\frac{\rho_{n}+c_{gn}\cos(\theta_{n})}{\sqrt{\rho_{n}^{2}+c_{gn}^{2}+2\rho_{n}c_{gn}\cos(\theta_{n})}}+\frac{\rho_{n}-c_{gn}\cos(\theta_{n})}{\sqrt{\rho_{n}^{2}+c_{gn}^{2}-2\rho_{n}c_{gn}\cos(\theta_{n})}}\\ & \;-\frac{d_{M\eta }c_{gn}\left[\cos(\theta_{n}-\psi_{M\eta })(c_{gn}+\rho_{n}\cos(\theta_{n}))-cos(\psi_{M\eta })(\rho_{n}+c_{gn}\cos(\theta_{n}))\right]}{{\left(\sqrt{\rho_{n}^{2}+c_{gn}^{2}+2\rho_{n}c_{gn}\cos(\theta_{n})}\right)}^{3}}] \end{array}$$

Due to the fact that the range *d_Mη_* is much smaller than polar range *ρ_n_* for the far-filed OS-BFSAR imaging processing, the third term in Equation (15) is approximated to zeros, so it can be neglected in general. Then, Equation (15) can be approximated as:

(16) $$\begin{array}{cc} k_{\rho_{n}} & \approx \frac{f}{c_{0}}\left[\frac{\rho_{n}+c_{gn}\cos(\theta_{n})}{\sqrt{\rho_{n}^{2}+c_{gn}^{2}+2\rho_{n}c_{gn}\cos(\theta_{n})}}+\frac{\rho_{n}-c_{gn}\cos(\theta_{n})}{\sqrt{\rho_{n}^{2}+c_{gn}^{2}-2\rho_{n}c_{gn}\cos(\theta_{n})}}\right]\\ & \approx \frac{f}{c_{0}}\cdot H(\delta_{n},\theta_{n}) \end{array}$$

Let *δ_n_* be defined as the ratio of *c_n_* to *ρ_n_* (i.e., *δ_n_* = *c_n_*/*ρ_n_*), and then the function *H*(*δ_n_*,*θ_n_*) is:

(17) $$H(\delta_{n},\theta_{n})=\frac{1+\delta_{n}\cos(\theta_{n})}{\sqrt{1+\delta_{n}^{2}+2\delta_{n}\cos(\theta_{n})}}+\frac{1-\delta_{n}\cos(\theta_{n})}{\sqrt{1+\delta_{n}^{2}-2\delta_{n}\cos(\theta_{n})}},\;\delta_{n}\geq 0$$

**Figure 4:**
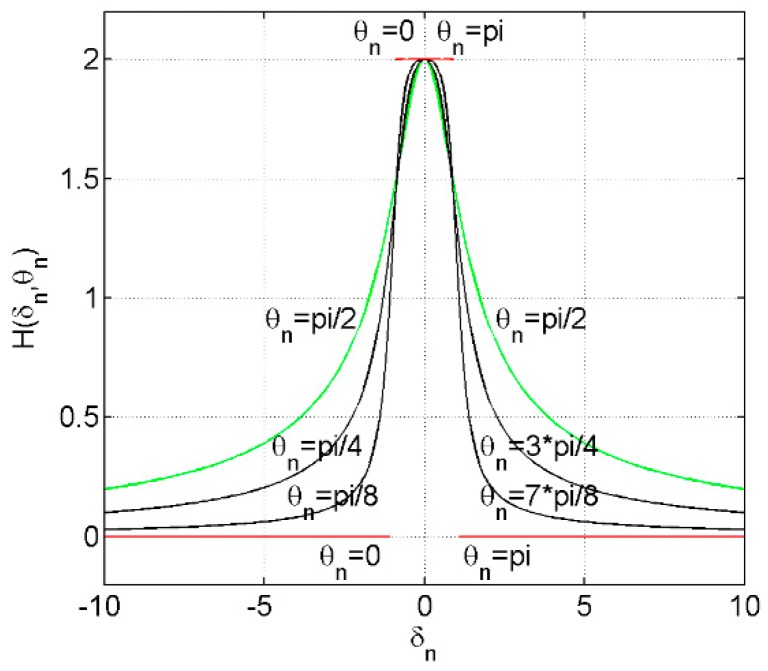
Function *H*(*δ_n_*,*θ_n_*) for different values of the angle *θ_n_*.

Figure 4 gives the plot of the function *H*(*δ_n_*,*θ_n_*) for different values of the angle *θ_n_*.

From Figure 4, we can clearly find that the minimum and maximum of the function *H*(*δ_n_*,*θ_n_*) are given by:

(18) $$\begin{array}{cc} H_{\max}(\delta_{n},\theta_{n}) & =\left\{ \begin{array}{ll} H(\delta_{n},0)\;\mathrm{or}\;H(\delta_{n},\pi ) & ,0\leq \delta_{n}\leq 1\\ H(\delta_{n},\pi /2) & ,\delta_{n}>1 \end{array}\right.\\ & =\left\{ \begin{array}{ll} 2 & ,0\leq \delta_{n}\leq 1\\ \frac{2}{\sqrt{1+\delta_{n}^{2}}} & ,\delta_{n}>1 \end{array}\right. \end{array}$$

and:

(19) $$\begin{array}{cc} H_{\min}(\delta_{n},\theta_{n}) & =\left\{ \begin{array}{ll} H(\delta_{n},\pi /2) & ,0\leq \delta_{n}\leq 1\\ H(\delta_{n},0)\;\mathrm{or}\;H(\delta_{n},\pi ) & ,\delta_{n}>1 \end{array}\right.\\ & =\left\{ \begin{array}{ll} \frac{2}{\sqrt{1+\delta_{n}^{2}}} & ,0\leq \delta_{n}\leq 1\\ 0 & ,\delta_{n}>1 \end{array}\right. \end{array}$$

Based on the maximum and minimum of the radar frequency *f*, the bound of the wavenumber $k_{\rho_{n}}$ can be given by:

(20) $$\frac{f_{\min}}{c_{0}}H_{\min}(\delta_{n},\theta_{n})\leq k_{\rho_{n}}\leq \frac{f_{\max}}{c_{0}}H_{\max}(\delta_{n},\theta_{n})$$

Then, the bandwidth of *I_FTn_*$\left(k_{\rho_{n}},\;k_{\theta_{n}}\right)$ with respect to the wavenumber $k_{\rho_{n}}$ is given by:

(21) $$B(k_{\rho_{n}})=\left\{ \begin{array}{ll} \frac{2}{c_{0}}(f_{\max}-f_{\min}/\sqrt{1+\delta_{n}^{2}}) & ,0\leq \delta_{n}\leq 1\\ \frac{2f_{\max}}{c_{0}\sqrt{1+\delta_{n}^{2}}} & ,\delta_{n}>1 \end{array}\right.$$

Finally, the sampling requirement for the polar range *ρ_n_* is therefore derived by:

(22) $$\Delta \rho_{n}\leq \frac{1}{B(k_{\rho_{n}})}=\left\{ \begin{array}{ll} \frac{c_{0}\sqrt{1+\delta_{n}^{2}}}{2(\sqrt{1+\delta_{n}^{2}}f_{\max}-f_{\min})} & ,0\leq \delta_{n}\leq 1\\ \frac{c_{0}\sqrt{1+\delta_{n}^{2}}}{2f_{\max}} & ,\delta_{n}>1 \end{array}\right.$$

For the monostatic SAR system with the co-located transmitter and receiver, by setting *δ_n_*= 0, the sampling requirement of the polar range used in the monostatic ETDA can be simplified as:

(23) $$\Delta \rho_{n}\leq \frac{c_{0}}{2(f_{\max}-f_{\min})}$$

which is the same with the equation derived and given in [36].

Similarly, based on the Equations (10), (12) and (14), the wavenumber $k_{\theta_{n}}$ is calculated by:

(24) $$\begin{array}{cc} k_{\theta_{n}} & =\frac{f}{c_{0}}\frac{\partial \left(R(\eta ,\rho_{n},\theta_{n})\right)}{\partial \theta_{n}}\\ & =\frac{f}{c_{0}}\frac{\partial }{\partial \theta_{n}}\left[R_{Mgn}+\;R_{Sgn}-d_{M\eta }\frac{c_{gn}\cos(\psi_{M\eta })+\rho_{n}\cos(\theta_{n}-\psi_{M\eta })}{\sqrt{\rho_{n}^{2}+c_{gn}^{2}+2\rho_{n}c_{gn}\cos(\theta_{n})}}\right] \end{array}$$

Since the first term in Equation (24) is nearly a constant with respect to the polar angle *θ_n_*, the first derivative of the first term with respect to polar angle *θ_n_* is zero, so the wavenumber $k_{\theta_{n}}$ is approximated by:

(25) $$\begin{array}{cc} k_{\theta_{n}} & =\frac{fd_{M\eta }\rho_{n}}{c_{0}}\frac{\left[\rho_{n}\sin(\theta_{n}-\psi_{M\eta })-c_{gn}\sin(\psi_{M\eta })\right]\left[\rho_{n}+c_{gn}\cos(\theta_{n})\right]}{{\left(\sqrt{\rho_{n}^{2}+c_{gn}^{2}+2\rho_{n}c_{gn}\cos(\theta_{n})}\right)}^{3}}\\ & \approx \frac{fd_{M\eta }\rho_{n}}{c_{0}}\frac{\left[\rho_{n}\sin(\theta_{n}-\psi_{M\eta })-c_{gn}\sin(\psi_{M\eta })\right]\left[\rho_{n}+c_{gn}\cos(\theta_{n})\right]}{\left[\rho_{n}^{2}+c_{gn}^{2}+2\rho_{n}c_{gn}\cos(\theta_{n})\right]\sqrt{\rho_{n}^{2}+c_{gn}^{2}\cos^{2}(\theta_{n})+2\rho_{n}c_{gn}\cos(\theta_{n})}}\\ & \approx \frac{fd_{M\eta }\rho_{n}}{c_{0}}\frac{\left[\rho_{n}\sin(\theta_{n}-\psi_{M\eta })-c_{gn}\sin(\psi_{M\eta })\right]}{\left[\rho_{n}^{2}+c_{gn}^{2}+2\rho_{n}c_{gn}\cos(\theta_{n})\right]}\\ & \approx \frac{fd_{M\eta }\left[\sin(\theta_{n}-\psi_{M\eta })-\delta_{n}\sin(\psi_{M\eta })\right]}{c_{0}[1+\delta_{n}^{2}+2\delta_{n}\cos(\theta_{n})]} \end{array}$$

In this paper, to find the bound of the wavenumber $k_{\theta_{n}}$ in Equation (25), we can investigate it in some extreme cases, i.e., the polar angle has such values *θ_n_* = 0, *θ_n_* = *π*/2 and *θ_n_* = *π*. As the cases *θ_n_* = 0 and *θ_n_* = *π* are considered, Equation (25) becomes:

(26) $$k_{\theta_{n}}(\theta_{n}=0)=\frac{-fd_{M\eta }\sin(\psi_{M\phi })}{c_{0}(1+\delta_{n})}$$

and:

(27) $$k_{\theta_{n}}(\theta_{n}=\pi )=\frac{fd_{M\eta }\sin(\psi_{M\eta })}{c_{0}(1-\delta_{n})}$$

Thus, the bound of the wavenumber $k_{\theta_{n}}$ in Equations (26) and (27) can be simply found from the values of the factors *f*, *d_Mη_* and sin(*ψ_Mη_*), which can be expressed as:

(28) $$-\frac{f_{\max}d_{Mn}}{c_{0}(1+\delta_{n})}\leq k_{\theta_{n}}(\theta_{n}=0)\leq \frac{f_{\max}d_{Mn}}{c_{0}(1+\delta_{n})}$$

and:

(29) $$-\frac{f_{\max}d_{Mn}}{c_{0}\left|1-\delta_{n}\right|}\leq k_{\theta_{n}}(\theta_{n}=\pi )\leq \frac{f_{\max}d_{Mn}}{c_{0}\left|1-\delta_{n}\right|}$$

where *d_Mn_* is the maximum of the length *d_Mη_*.

Similarly, when the case *θ_n_* = *π*/2 is considered, Equation (25) becomes:

(30) $$\begin{array}{cc} k_{\theta_{n}}(\theta_{n}=\pi /2) & =\frac{fd_{M\eta }[\cos(\psi_{M\eta })-\delta_{n}\sin(\psi_{M\eta })]}{c_{0}(1+\delta_{n}^{2})}\\ & =\frac{fd_{M\eta }g(\psi_{M\eta })}{c_{0}(1+\delta_{n}^{2})} \end{array}$$

where g(*ψ_Mη_*) is the trigonometric function, i.e., g(*ψ_Mη_*) = cos(*ψ_Mη_*) – *δ_n_*sin(*ψ_Mη_*). The extremum of this trigonometric function can be estimated with roots of its first derivative with respect to the angle *ψ_Mη_*, i.e.:

(31) $$\frac{\partial \left[g(\psi_{M\phi })\right]}{\partial \psi_{M\phi }}=0$$

Then, we have:

(32) $$\psi_{M\phi }=\mathrm{arc}\text{​}\tan(-\delta_{n})$$

Therefore, the bound of the function g(*ψ_Mη_*) is given by:

(33) $$-\sqrt{1+\delta_{n}^{2}}\leq g(\psi_{M\phi })\leq \sqrt{1+\delta_{n}^{2}}$$

As a result, the bound of the wavenumber $k_{\theta_{n}}$ in Equation (30) is obtained as:

(34) $$-\frac{f_{\max}d_{Mn}}{c_{0}\sqrt{1+\delta_{n}^{2}}}\leq k_{\theta_{n}}(\theta_{n}=\pi /2)\leq \frac{f_{\max}d_{Mn}}{c_{0}\sqrt{1+\delta_{n}^{2}}}$$

Therefore, the bandwidth of *I_FTn_*$\left(k_{\rho_{n}},\;k_{\theta_{n}}\right)\;$ with respect to the wavenumber $k_{\theta_{n}}$ for these above extreme cases can be given by:

(35) $$\left\{ \begin{array}{l} B(k_{\theta_{n}}(\theta_{n}=0))=\frac{2f_{\max}d_{Mn}}{c_{0}(1+\delta_{n})}\\ B(k_{\theta_{n}}(\theta_{n}=\pi ))=\frac{2f_{\max}d_{Mn}}{c_{0}\left|1-\delta_{n}\right|}\\ B(k_{\theta_{n}}(\theta_{n}=\pi /2))=\frac{2f_{\max}d_{Mn}}{c_{0}\sqrt{1+\delta_{n}^{2}}} \end{array}\right.$$

From Equation (35), it can be seen that $B\left(k_{\theta_{n}}\left(\theta_{n}=\pi \right)\right)\geq B\left(k_{\theta_{n}}\left(\theta_{n}=\;0\right)\right)$ and $B\left(k_{\theta_{n}}\left(\theta_{n}=\pi \right)\right)\geq B\left(k_{\theta_{n}}\left(\theta_{n}=\;\pi /2\right)\right)$, thus the sampling requirement for the polar angle $\theta_{n}$ is derived from the bandwidth $B\left(k_{\theta_{n}}\left(\theta_{n}=\pi \right)\right)$, which is given by:

(36) $$\begin{array}{cc} \Delta \theta_{n} & \leq \frac{1}{B(k_{\theta_{n}}(\theta_{n}=\pi ))}\\ & =\frac{c_{0}\left|1-\delta_{n}\right|}{2f_{\max}d_{Mn}}=\frac{c_{0}\left|1-\delta_{n}\right|}{f_{\max}\sqrt{l_{Mn}^{2}+4\delta_{Mn,\max}^{2}}} \end{array}$$

where, *l_Mn_* is the length of the *n*-th subaperture along the Y axis direction, and *δ_Mn,max_* is the maximum of the range *δ_Mn_*. From Equation (36), it can be found that the efficiency of the proposed ETDA will reduce when the deviation from the ideal flight track becomes large in comparison with the bistatic DTDA.

For the monostatic SAR imaging processing, by setting *δ_n_*= 0 and *d_Mn_* = 2*d_n_* (*d_n_* is length of the *n*-th subaperture for the monostatic SAR), the polar angle sampling requirement used in the monostatic ETDA can be simplified as:

(37) $$\Delta \theta_{n}\leq \frac{c_{0}}{2f_{\max}\sqrt{l_{Mn}^{2}+4\delta_{Mn,\max}^{2}}}$$

which is also the same as the equation derived and given in [36].

### 3.3. Algorithm Implementation

**Figure 5:**
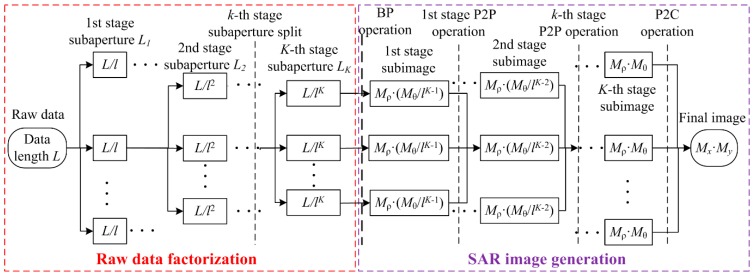
Implementation of the proposed ETDA for the OS-BFSAR imaging processing.

The implementation of the proposed ETDA for the OS-BFSAR imaging processing is similar to that of the ETDA for the traditional one-stationary BSSAR (OS-BSSAR) imaging processing given in [46], but it calculates the subimages on the polar grids in the ground plane instead of the elliptical polar grids in the slant-range plane, which can be referenced to the positions of both moving and stationary radars. Figure 5 shows the implementation of the proposed ETDA for the OS-BFSAR imaging processing, and it contains two parts: the raw data factorization and SAR image generation, which are marked by the dashed rectangles with the different colors. The former includes the factorization of the received echo data and moving radar track data (i.e., the moving radar synthetic aperture), while the latter includes calculating the polar grids and Cartesian grid, performing the BP on the polar grids, interpolating the polar subimages to the polar subimages (P2P), and interpolating the polar subimages to the Cartesian image (P2C).

Similarly, the proposed ETDA improves the imaging efficiency by dividing the full synthetic aperture of the moving radar with *L* aperture positions. In other words, the full synthetic aperture is split recursively *K* times or stages by a factor of *F_k_* in the *k*-th (1 ≤ *k* ≤ *K*) stage, and until $\prod_{k=1}^{K}F_{k}$ subapertures of the size $L_{K}=L/\prod_{k=1}^{K}F_{k}$ are finally reached. Then, we have:

(38) $$L=L_{K}\cdot \prod_{k=1}^{K}F_{k}k=1,\cdot \cdot \cdot ,K,$$

where *F_k_* is defined as the reduction in the number of aperture positions *F_k_* for the moving radar during the *k*-th processing stage. For simplification, we assume that there is a constant factorization of the full aperture positions of the moving radar during all processing stages, i.e., *F_k_*= *l* for all *k* and K = log*_l_*(*L*/*L_K_*). Therefore, Equation (38) can be simplified as:

(39) $$L=L_{K}\cdot l^{K}$$

In the first stage, the full synthetic aperture of the moving radar is split into *l^K^* small subapertures, which requires a split of the range-compressed echo data similarly. Taking the *n*-th subaperture imaging processing in the first stage for example, the polar grids of the subimage are defined according to the derived sampling requirements. The regular BP for the *n*-th subaperture is performed on the corresponding polar grids over an elliptical mapping, and then accumulated coherently to generate the coarse polar subimage. The parameters *ρ_n_*, *θ_n_*, *ρ_np_*, *θ_np_*, *η_n_* and *T_n_* in Equation (8) are rewritten as $\rho_{n}^{1},\;\theta_{n}^{1},\;\rho_{np}^{1},\;\theta_{np}^{1},\;\eta_{n}^{1}\;and\;T_{n}^{1}$, respectively. Hence, the *n*-th polar subimage at the first stage is given by:

(40) $$I_{n}^{1}\left(\rho_{n}^{1},\theta_{n}^{1}\right)=\int_{\eta_{n}^{1}-T_{n}^{1}/2}^{\eta_{n}^{1}+T_{n}^{1}/2}\sigma_{P}p_{rc}\left[\frac{B\left(R(\eta ,\rho_{n}^{1},\theta_{n}^{1})-R(\eta ,\rho_{nP}^{1},\theta_{nP}^{1})\right)}{c_{0}}\right]\exp\left[\frac{j2\pi fR(\eta ,\rho_{n}^{1},\theta_{n}^{1})}{c_{0}}\right]d\eta$$

The second stage is a recursive procedure. For *k*-th stage (2 ≤ *k* ≤ *K*), the polar subimages at the *k*-th stage are generated from the polar subimages formed in the (*k* − 1)-th stage. First, every *l* subapertures of the moving radar at the (*k* − 1)-th stage are combined into a new subaperture at the *k*-th stage. The origin of the new polar grid is defined as the projection of the center point of the link line between the stationary radar and the new subaperture center of the moving radar, and then the new polar grids ($\rho_{q}^{k},\theta_{q}^{k})$ are defined according to the derived sampling requirements. To generate the *q*-th polar subimages at the *k*-th stage, every *l* corresponding polar subimages at the (*k* − 1)-th stage are interpolated into the polar grids ($\rho_{q}^{k}$,$\theta_{q}^{k})$ and then accumulated coherently, which is:

(41) $$I_{q}^{k}(\rho_{q}^{k},\theta_{q}^{k})=\sum_{p=1+(q-1)l}^{ql}I_{p}^{\left(k-1\right)}(\rho_{p,cor}^{k-1},\theta_{p,cor}^{k-1})$$

where, $I_{q}^{k}$ is the *q*-th polar subimage in the *k*-th stage, and $I_{q}^{k-1}$ is the *p*-th polar subimage at the (*k* − 1)-th stage. ($\rho_{p,cor}^{k-1},\theta_{p,cor}^{k-1})$ is the corresponding position of the polar grid ($\rho_{q}^{k},\theta_{q}^{k})$ in the polar subimage $I_{q}^{k-1}$. However, in the practice terms, the position ($\rho_{p,cor}^{k-1},\theta_{p,cor}^{k-1})$ may not be just a discrete sample position in the polar subimage $I_{q}^{k-1}$. According to [40,41], the values of the position ($\rho_{p,cor}^{k-1},\theta_{p,cor}^{k-1})$ in the polar subimages $I_{q}^{k-1}$ should be interpolated from that of its surrounding samples in l polar subimages $I_{p}^{k-1}$ by the different interpolation methods [50]. Please note that, before the interpolation is operated, the upsampling of the data of the polar subimages $I_{q}^{k-1}$ in the polar range and polar angle is usually required in the proposed ETDA, since it will directly affect the final SAR image quality [50].

According to [46], if the sampling requirements for the polar grids are satisfied for all processing stages, it is found that the grid interpolation is the main source of error in the proposed ETDA, and is also the computational bottleneck of the proposed algorithm. It is well known that the quality of the SAR image is a trade-off between the imaging precision and efficiency. Fortunately, the high precision interpolators used in the ETDA can be easy to obtain in [50]. The two dimensional linear interpolations are used in the proposed ETDA due to their high efficiency. The low-pass linear interpolation is used in the polar angle since the angular signal is low-pass, and the band-pass linear interpolation is used in the polar range since the transmitted signal is band-pass [46]. Of course, there are also other interpolation methods. For example, the cubic spline interpolation can be used in the polar angle and the fast Fourier transform (FFT) and inverse FFT (IFFT) in combination with the zero padding can be used in the polar range, which is another alternative.

In the final stage, the final Cartesian SAR image is generated from all polar subimages at the *K*-th stage. First, all aperture positions of the moving radar are combined into a whole aperture. Then, the Cartesian grid (*x,y*) is defined according to the resolutions of the final image. Finally, *l* polar subimages at the *K*-th stage are interpolated into the Cartesian grid, and then summed coherently to reconstruct the Cartesian OS-BFSAR image, which is given by:

(42) $$I(x,y)=\sum_{p=1}^{l}I_{p}^{K}(\rho_{p,cor}^{K},\theta_{p,cor}^{K)})$$

where *I*(*x,y*) is the Cartesian OS-BFSAR image and $\left(\rho_{p,cor}^{K},\theta_{p,cor}^{K}\right)$ is the corresponding position of the Cartesian grid (*x,y*) in the *p*-th subimage $I_{q}^{K}$ at the *K*-th stage.

### 3.4. Computational Load

Similar to the ETDA for the traditional OS-BSSAR imaging processing presented in [46], the computational load (number of operations) of the proposed ETDA for the OS-BFSAR imaging processing mainly includes the operation number of calculating the polar grids and Cartesian grid, the operation number of performing the BP on the polar grids, the operation number of the P2P interpolation, and the operation number of the P2C interpolation during all processing stages.

Provided that the final Cartesian SAR image has the size of *M_x_* and *M_y_* in the X and Y axes directions, respectively, while the polar subimage at the *K*-th stage has the size of *M_ρ_* and *M_θ_* in the polar range and polar angle directions, respectively. For the first stage, the size of the polar subimage is assumed to be *M_ρ_*·(*M_θ_*/*l^K^*^–1^), and therefore the operation number of calculating the polar grids *O*_1*,Grid*_ at this stage is given by:

(43) $$\begin{array}{cc} O_{1,Grid} & =l^{K}\cdot M_{\rho }\cdot (M_{\theta }/l^{K-1})\\ & =lM_{\rho }M_{\theta } \end{array}$$

Similarly, the operation number of performing the BP on the polar grids *O_BP_* at this stage can be calculated by:

(44) $$\begin{array}{cc} O_{BP}\; & =l^{K}\cdot (L/l^{K})\cdot M_{\rho }(M_{\theta }/l^{K-1})\\ & =(L/l^{K-1})M_{\rho }M_{\theta } \end{array}$$

Thus, the total operation number of the imaging processing at first stage is:

(45) $$\begin{array}{cc} O_{1} & =O_{1,Grid}+O_{BP}\\ & =(l+L/l^{K-1})M_{\rho }M_{\theta } \end{array}$$

For the *k*-th stage (2 ≤ *k* ≤ *K*), the size of the polar subimage is assumed to *M_ρ_*·(*M_θ_*/*l^K^*^–*k*^), thus the operation number of calculating the polar grids *O*_2*,Grid*_ at this stage is given by:

(46) $$\begin{array}{cc} O_{2,Grid} & =\sum_{k=2}^{K}l^{K-k+1}M_{\rho }(M_{\theta }/l^{K-k})\\ & =(K-1)lM_{\rho }M_{\theta } \end{array}$$

Similarly, the operation number of interpolating the polar subimages into the polar subimages *O_P_*_2*P*_ in this stage is given by:

(47) $$\begin{array}{cc} O_{P2P} & =\sum_{k=2}^{K}l^{K-k+1}\cdot l\cdot M_{\rho }(M_{\theta }/l^{K-k})\\ & =(K-1)l^{2}M_{\rho }M_{\theta } \end{array}$$

Therefore, the total operation number of the imaging processing at this stage is:

(48) $$\begin{array}{cc} O_{2} & =O_{2,Grid}+O_{P2P}\\ & =(K-1)l(1+l)M_{\rho }M_{\theta } \end{array}$$

For the final stage, the size of the Cartesian image is assumed to *M_x_*·*M_y_*, thus the operation number of calculating the Cartesian grid *O*_3*,Grid*_ in this stage is given by:

(49) $$O_{3,Grid}=M_{x}M_{y}$$

Thus, the operation number of interpolating the polar subimages into the Cartesian image *O_P_*_2*C*_ is:

(50) $$\begin{array}{cc} O_{P2C} & =l\cdot M_{x}\cdot M_{y}\\ & =lM_{x}M_{y} \end{array}$$

Thus, the total operation number of the imaging processing at the final stage is:

(51) $$\begin{array}{cc} O_{3} & =O_{3,Grid}+O_{P2C}\\ & =(1+l)M_{x}M_{y} \end{array}$$

As a result, the total operation number of the proposed ETDA is given by:

(52) $$\begin{array}{cc} O_{\mathrm{ETDA}} & =O_{1}+O_{2}+O_{3}\\ & =M_{x}M_{y}(1+l)+M_{\rho }M_{\theta }\left(Kl+(K-1)l^{2}+L/l^{K-1}\right) \end{array}$$

We assume that *M_ρ_* = *μ_ρ_M_x_* and *M_θ_* = *μ_θ_M_y_*, and then Equation (52) can be approximated as:

(53) $$O_{\mathrm{ETDA}}=M_{x}N_{y}\left[1+l+\mu_{\rho }\mu_{\theta }\left(Kl+(K-1)l^{2}+L/l^{K-1}\right)\right]$$

Analogously, the operation number of the bistatic DTDA can be calculated by:

(54) $$\begin{array}{cc} O_{\mathrm{DTDA}} & =O_{1,Grid}+O_{BP}\\ & =M_{x}\cdot M_{y}+L\cdot M_{x}\cdot M_{y}\\ & =\;M_{x}M_{y}(1+L) \end{array}$$

The speed-up factor of the proposed ETDA with respect to the bistatic DTDA is defined as:

(55) $$\begin{array}{cc} \kappa_{\;\mathrm{ETDA}}= & \frac{O_{\mathrm{DTDA}}}{O_{\mathrm{ETDA}}}\\ & =\frac{1+L}{1+l+\mu_{\rho }\mu_{\theta }\left(Kl+(K-1)l^{2}+L/l^{K-1}\right)} \end{array}$$

**Figure 6:**
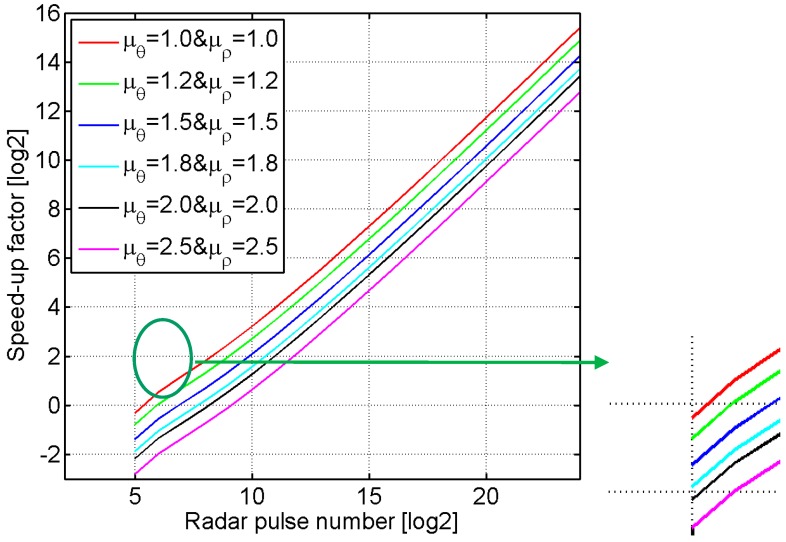
The logarithm (base 2) of the speed-up factor of the proposed ETDA for the different values of the factors *μ_θ_* and *μ_ρ_*.

The value of the speed-up factor *κ*_EDTA_ is determined by factors *L*, *l*, *K*, *μ_θ_* and *μ_ρ_*, and the values of factors *μ_θ_* and *μ_ρ_* are both larger than or equal to one in general. When the radar pulse number *L* (i.e., the length of the synthetic aperture of the moving radar) increases, only the factor *K* in Equation (55) increases in *K* = log*_l_*(*L*/*L_K_*), where the factors *l*, *μ_θ_* and *μ_ρ_* are assumed to be the constant values. Figure 6 shows the logarithm (base 2) of the speed-up factor with respect to the radar pulse number *L* for the different values of factors *μ_θ_* and *μ_ρ_*, where *L_K_* = 16 and *l* = 4 are assumed. From Figure 6, it is clearly found that the logarithm values of these speed-up factors marked by different colors are almost directly proportional to log_2_*L* in some range.

The larger the product of factors *μ_θ_* and *μ_ρ_*, the smaller the value of the speed-up factor. The red line indicates the speed-up factor on the condition *μ_θ_* = *μ_ρ_*= 1, so the value of the speed-up factor is almost larger than one for all the synthetic apertures. However, in the practical SAR imaging, the factors *μ_θ_* and *μ_ρ_* are both larger than one, and therefore, the value of speed-up factor is smaller than one for the small synthetic aperture (see the zoom image in the green ellipse in Figure 6). The reason may be that the proposed ETDA spends more time on the calculation and interpolation of polar grids than that of the bistatic DTDA does. Comparing with the bistatic DTDA, the proposed ETDA has a good acceleration for the moderate and high resolution OS-BFSAR imaging processing, but doesn’t offer any acceleration for the low resolution OS-BFSAR imaging processing.

## 4. Experimental Results

In this section, in order to verify the validity of the proposed ETDA for the OS-BFSAR imaging processing, experimental results based on both simulated and measured data are shown and analyzed to compare the performance of the proposed ETDA with that of the bistatic DTDA. The SAR scene reconstructed using the bistatic DTDA is used as the reference for the comparison, since this algorithm is the most accurate method for the imaging processing without any approximation in theory. The simulated OS-BFSAR data of the scene including several discrete scattering targets and the measured OS-BFSAR data acquired by an OS-BFSAR experiment at P-band are processed by the two algorithms, and then imaging results are illuminated in this section.

### 4.1. Simulated Data Results

**Figure 7:**
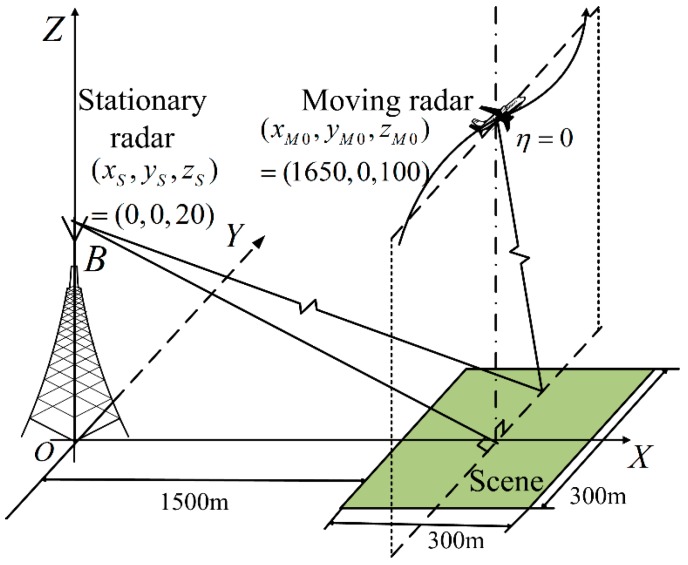
Imaging geometry of the simulated OS-BFSAR system.

**Figure 8:**
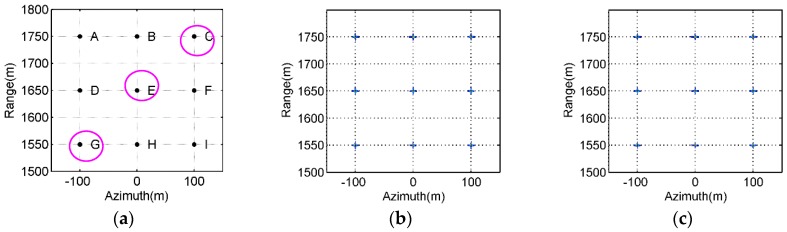
Imaging results of the simulated data obtained by the different algorithms. (**a**) Distribution of the point-like scatterers; (**b**) Bistatic DTAD; (**c**) Proposed ETDA.

The imaging geometry of the simulated OS-BFSAR system including the motion errors is the same as that in Figure 7. The parameters of the simulated OS-BFSAR system are shown in Table 1. The stationary radar is located on the top of a high tower, and its position is (0,0,20) m. The ideal flight track of the moving radar is parallel to the Y-axis direction, and its initial position is (1650,0,100) m at the slow time *η* = 0. In this OS-BFSAR system, both moving and stationary radars work in the forward-looking and spotlight mode. Suppose that the illuminating beam of the transmitter is always covered by that of the receiver to insure the synchronization of the OS-BFSAR system. Based on these parameters listed in Table 1, it is easy to compute that the moving radar synthetic aperture time *T_a_* is 6.5 s. The motion error is added to the ideal flight track of the moving radar. The motion error in the X axis direction is *δM_x_* = 5sin(2*π*(1/*T_a_*)*η*) + 0.3*η*, the motion error in the Y axis direction is *δM_y_* = 2sin(2*π*(0.3/*T_a_*)*η*) + 0.1*η* and the motion error in the Z axis direction is *δM_z_* = 3sin(2*π*(0.5/*T_a_*)*η*) + 0.2*η*. The simulated ground scene contains nine discrete point-like scatterers labeled as A–I in 3 rows and 3 columns, which are equally spaced in an area with the size of 300 m × 300 m in the azimuth and range directions, respectively. And, the point-like scatterer E is located at the scene center position (1650,0,0) m. Both the range and azimuth intervals of all point-like scatterers are 100 m in the ground plane, and the radar cross sections of all point-like scatterers are assumed and normalized to be 1 m^2^ for simplification. The samples of the Cartesian image grids are assumed to be 0.8 m × 0.6 m in the azimuth and range directions, respectively. The distribution of the point-like scatterers is shown in Figure 8a. For evaluation purposes, the effects of the radio frequency interference (RFI), jamming, thermal noise, clutter, multipath, incidence/ reflection angles, local reflection and so on are not considered in this simulation. Moreover, in order to observe the performance of the reconstructed SAR images more clearly and to make a fair comparison, no weighting function or sidelobe control approach is used in this simulation.

Figure 8b,c give the imaging results of the simulated SAR scene obtained by the bistatic DTDA and proposed ETDA, respectively. From Figure 8b, it is seen that the simulated SAR scene is well reconstructed by the DTDA, and all point-like scatterers appear in the SAR image as points. As observed from Figure 8c, all point-like scatterers in the simulated SAR scene are also well focused by the proposed ETDA, and also appear in the SAR image as points. It can be found that the focusing of all point-like scatterers in Figure 8c is very similar to that in Figure 8b. Visually, there is nearly no difference between SAR images given in Figure 8b,c, which indicates that the proposed ETDA is very effective for the OS-BFSAR imaging processing.

**Figure 9:**
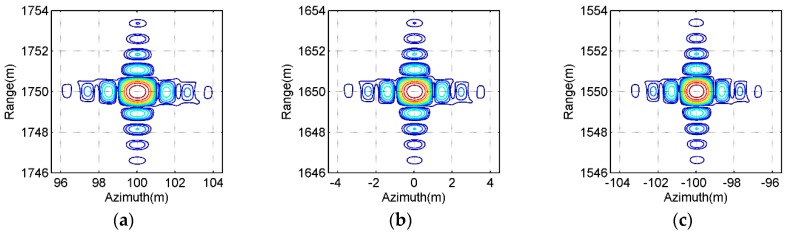
Contours of the imaging results obtained by the bistctic DTDA for all selected point-like scatterers in Figure 8a. (**a**) Scatterer C; (**b**) Scatterer E; (**c**) Scatterer G.

**Figure 10:**
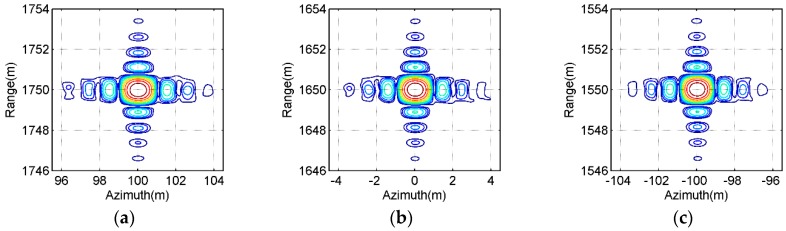
Contours of imaging results obtained by the proposed ETDA for all selected point-like scatterers in Figure 8a. (**a**) Scatterer C; (**b**) Scatterer E; (**c**) Scatterer G.

Figure 9 and Figure 10 show the contours of the imaging results of the point-like scatterers C, E and G labeled in Figure 8a, which are extracted from Figure 8b,c, respectively. From Figure 9, we may observe the general ultrawideband (UWB) features of all selected point-like scatterers, such as the orthogonal and nonorthogonal sidelobes, etc. By observing Figure 10, it is seen that the contours of the imaging results of all selected point-like scatterers is very similar to those shown in Figure 9. Also, the point targets with the typical features of a point-like scatterer illuminated by a UWB SAR system can be observed in the SAR image given in Figure 10. However, the focusing quality of all selected point-like scatterers in Figure 10 is slightly degraded in comparison to the reference one in Figure 9, since there may be still small phase error caused by interpolations in the proposed ETDA. The effects are invisible at the high contour levels and some small influences is observed at lower contour levels, i.e., only the sidelobes of focused point-like scatterers suffer from the effect of phase errors. The phase errors are relatively small (usually smaller than or equal to *π*/8) and therefore do not strongly affect the peak signal level and itssurrounding of the focused point-like scatterers.

**Figure 11:**
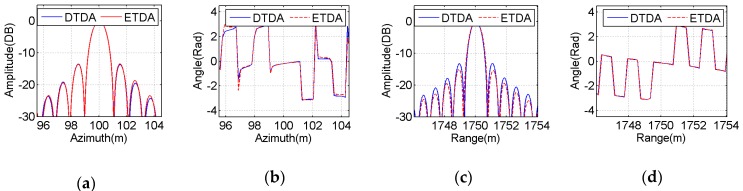
Comparison between the focused results obtained by the bistatic DTDA and proposed ETDA for the point-like scatterer C. (**a**) Azimuth amplitude; (**b**) Azimuth phase; (**c**)Range amplitude; (**d**) Range phase.

**Figure 12:**
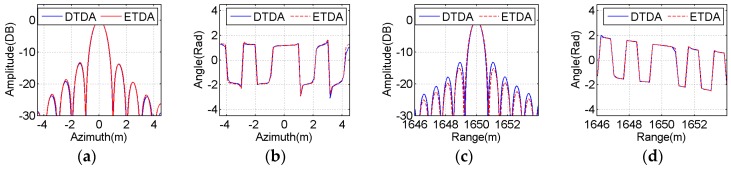
Comparison between the focused results obtained by the bistatic DTDA and proposed ETDA for the point-like scatterer E. (**a**) Azimuth amplitude; (**b**) Azimuth phase; (**c**) Range amplitude; (**d**) Range phase.

**Figure 13:**
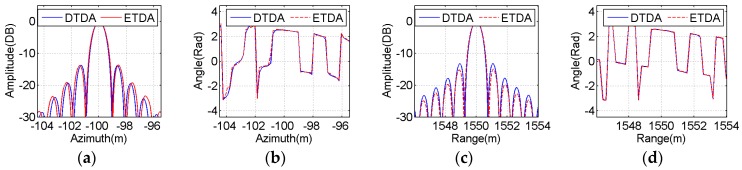
Comparison between the focused results obtained by the bistatic DTDA and proposed ETDA for the point-like scatterer G. (**a**) Azimuth amplitude; (**b**) Azimuth phase; (**c**)Range amplitude; (**d**) Range phase.

To perform a more illustrative evaluation, both amplitude and phase profiles of the imaging results of all selected point-like scatterers in the azimuth and range directions are extracted from Figure 9 and Figure 10 and then plotted in Figure 11, Figure 12 and Figure 13. The blue solid line indicates the profiles with the bistatic DTDA, while the red dashed line represents the profiles with the proposed ETDA. As observed from the images in Figure 11, Figure 12 and Figure 13, both amplitude and phase profiles of the imaging results of all selected point-like scatterers by the two algorithms are very similar. In the mainlobe areas of the amplitude profiles for all selected point-like scatterers, it is almost impossible to see any difference between the two algorithms. However, in the sidelobe areas of the amplitude profiles, there is still slight difference between the two algorithms, which may be caused by the effects of the phase errors in the proposed ETDA. It might be clearly found that the similar performance can be observed in the phase profiles for all selected point-like scatterers. Therefore, such influences clearly show that the phase errors in the proposed ETDA may degrade slightly the focusing quality of the OS-BFSAR image compared with the bistatic DTDA.

To quantitatively analyze the imaging performance of the proposed ETDA in comparison to the bistatic DTDA, the quality measurements for the OS-BFSAR image can be calculated based on the amplitude profiles in Figure 11, Figure 12 and Figure 13, such as the spatial resolution, peak sidelobe ratio (PSLR) and integrated sidelobe ratio (ISLR). Spatial resolutions in the azimuth and range directions can be defined by −3 dB width of the mainlobe of the focused point-like scatterer, i.e., the distance between two points where the intensity is one half of the peak intensity.

PSLR can be defined by the ratio of the peak intensity in the sidelobe area to the peak intensity in the mainlobe area, and ISLR can be defined by the ratio of the energy of the sidelobe to that of the mainlobe. The results of these measured parameters are listed in Table 2. As observed from Table 2, it is found that all measured parameters obtained by the two algorithms are very similar. The resolutions in the azimuth and range directions of all selected point-like scatterers by the proposed ETDA become little worse than those of the bistatic DTDA. Fortunately, the PSLRs and ISLRs of all selected point-like scatterers by the proposed ETDA are very similar to those by the bistatic DTDA. Based on the above-mentioned analysis of the imaging results, we can conclude that the imaging result by the proposed ETDA is very similar to that by the bistatic DTDA.

In order to prove the imaging efficiency of the proposed ETDA, the operation number of the two imaging methods is estimated in this simulation case. The simulated data are processed by the two algorithms using the next parameters: *M_x_* = 500, *M_y_* = 375, *L* = 2^24^, *L_K_* = 16, *l* = 4, *μ_θ_* = *μ_ρ_* = 1.2, thus *K* = log*_l_*(*L*/*L_K_*) = 10. From Equation (55), the number of operations of the bistatic DTDA can be therefore calculated by *O*_DTDA_ = *M_x_M_y_*(1 + *L*) ≈ 3.15 × 10^12^, while the operation number of the proposed ETDA is given by *O*_ETDA_ = *M_x_N_y_*[1 + *l*+ *μ_ρ_μ_θ_*(K*l* + (*K*–1)*l*^2^ + *L/l^K^*^–1^)]) ≈ 1.05 × 10^8^. Then, the speed-up factor of the proposed ETDA to the bistatic DTDA is *κ*_ETDA_ = *O*_DTDA_/*O*_ETDA_ ≈ 3.01 × 10^4^, and the logarithm (base 2) of the speed-up factor *κ*_ETDA_ is about 14.87. Besides, the processing time of two algorithms is measured on the same condition, which depends directly on the interpolation method, upsampling factor and computational platform. Radar echo data is interpolated as follows: one dimension complex linear interpolation with the upsampling factor of 4 for the BP operation at the first stage of the proposed ETDA, as well as the bistatic DTDA; two dimension complex linear interpolation with the upsampling factor of 4 for the P2P and P2C operations at the successive stages of the proposed ETDA, since it has the high precision and efficiency and the well adaptability to the band-limited signals. They are programmed in the MATLAB version 7.10.0 on a Personal Computer (PC) with a 2.93 GHz Dual-Core Central Processing Unit (CPU) and a 2.00 GB Random Access Memory (RAM). The processing time of the bistatic DTDA and proposed ETDA are 665.8 s and 46.2 s, respectively. Compared with the bistatic DTDA, the imaging speed of the proposed ETDA is improved about 14.5 times, which corresponds with the theoretical results in Figure 6. According to [45], if the proposed ETDA is parallelized on the Graphic Processing Unit (GPU), the proposed ETDA will further accelerate the process in the OS-BFSAR imaging.

### 4.2. Measured Data Results

**Figure 14:**
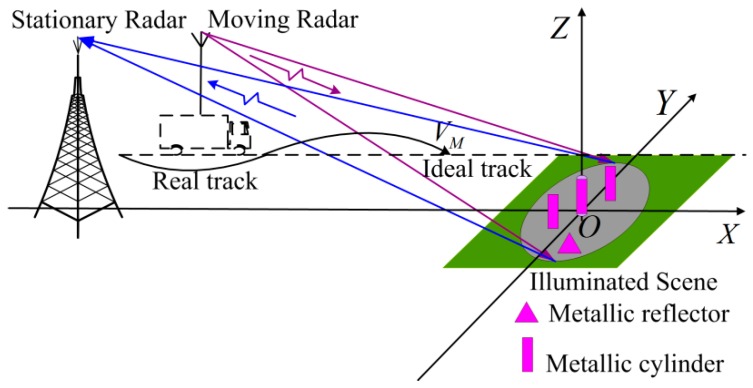
Imaging geometry of the real OS-BFSAR system.

**Figure 15:**
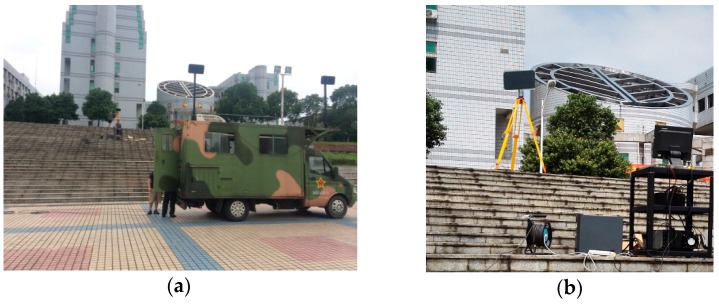
Real OS-BFSAR system. (**a**) Vehicle-based moving radar; (**b**) Ground-based stationary radar.

In order to further prove the feasibility of the proposed ETDA, the measured data acquired by a real OS-BFSAR system are processed by the two algorithms, and the imaging results are shown and analyzed. The imaging experiment of the OS-BFSAR system has been carried out at P-band in late 2015, which has the construction of the ground-based stationary radar to operate in conjunction with an existing vehicle-based monostatic SAR system. The imaging geometry of the real OS-BFSAR system is shown in Figure 14, and the real OS-BFSAR system is given in Figure 15. The stationary radar is located on the top of a tripod on a high position. The moving radar is fixed on the top of the vehicle in a square, and the dashed line is the ideal track of the vehicle, while the solid curve is its actual track. The vehicle-based moving radar continually illuminates the scene including several targets and transmits the chirp signal (P-band) in the forward-looking and side-looking modes, while the ground-based stationary radar illuminates the same region and receives the bistatic scattered signal of the scene. Both vehicle-based and ground-based antennas have a 3 dB azimuth beamwidth of approximately 35° to 50°, and a 3 dB elevation beamwidth of approximately 75° to 85°. To maintain the time synchronization of the OS-BFSAR system, the one pulse per second (1PPS) output signal from a global positioning system (GPS) receiver incorporated into the moving and stationary radars is used as a timing reference. Besides, to maintain the frequency synchronization the OS-BFSAR system, the voltage-controlled oscillator (VCO) generate a standard sine signal, after amplification and frequency conversion, this signal is transferred into a phase detector, and it is phase-detected by the 1PPS signal. The phase error from the phase detector is used to discipline the VCO, and the output signal from this in turn stabilizes the VCO via the digital phase-locked loop (DPLL). VCO produces coherent reference signals in the moving and stationary radars, and these are used to drive the frequency synthesizer to produce the various system signals. This technique can real-timing revise the time and frequency reference signals to satisfy system synchronization.

**Figure 16:**
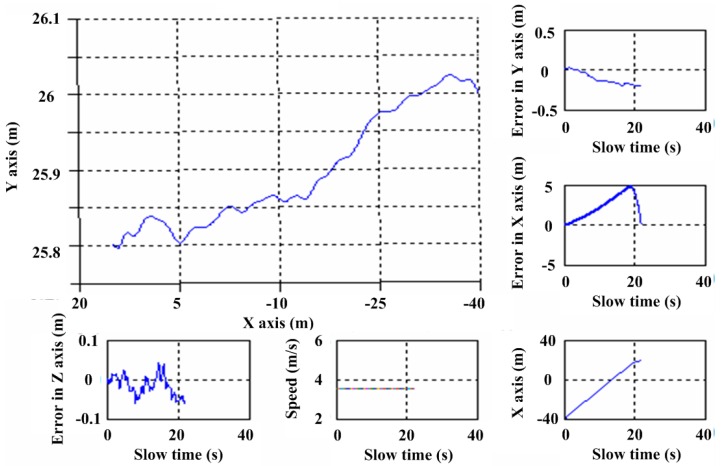
Position of the vehicle-based radar from the GPS data.

**Figure 17:**
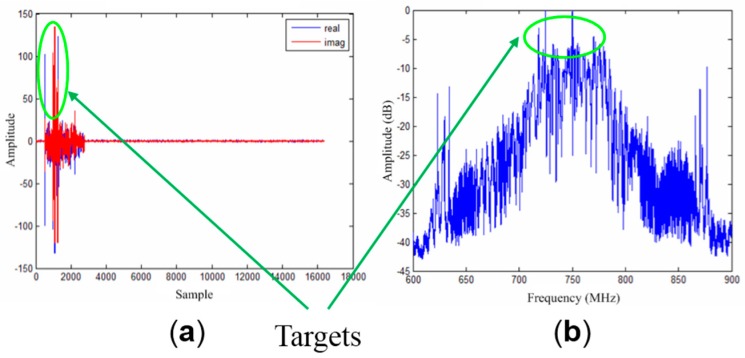
Bistatic scattered signal of the scene. (**a**) Bistatic scattered signal from one transmitted radar pulse, including the real (blue line) and imaginary (red line); (**b**) The sum of the frequency spectrum of the bistatic scattered signal from 100 transmitted radar pulses. Note that the scattered signal of the targets is in the green ellipses.

The acquisition parameters of the real OS-BFSAR system are shown in Table 3. Figure 16 gives the position of the vehicle-based radar from the GPS data, including the actual position in the X and Y axes directions, the motion errors in the X, Y and Z axes directions, and so on. The vehicle moves parallel to the X-axis direction with an average speed about 12.8 km/h (3.55 m/s), and the average height of the vehicle-based antenna is about 4 m. The position of the ground-based antenna is about (−54,0,6)m, and the initial position of the vehicle-based antenna is about (−40,26,4) m. As the scene being imaged must lie in the line-of-sight of the stationary radar, this constrained operation of the radar system to the imaging of test-sites within only a few decameters from the tripod. One data site was acquired for the imaging processing: the scene was a flat area, and its size is about 40 m in the X axis direction and 35 m in the Y axis direction. Various scattering targets, including three metallic cylinders and one metallic trihedral reflector were deployed at the test-site prior to this experiment, and their positions in the ground plane are about (5.5,5.5,0) m, (0,0,0) m, (−5.5,−5.5,0) m and (7.5,−17.5,0) m, respectively. As the vehicle-based radar system moves parallel to the X-axis direction, the bistatic measured data were naturally collected from a wide variety of the azimuth look direction. Figure 17 gives the bistatic scattered signal of the scene in the time domain and frequency domain. As observed from Figure 17, it can be clearly found that the scattered signal of the targets is very obvious (see the region in the green ellipses in Figure 17).

**Figure 18:**
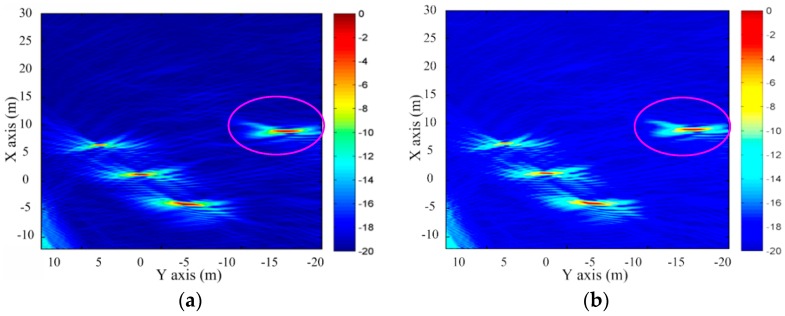
Imaging results of the measured data obtained by the different algorithms. (**a**) Bistatic DTAD; (**b**) Proposed ETDA.

Next, the measured data of the scene is processed by the bistatic DTDA and proposed ETDA. Before the imaging processing is performed by two methods, the measured data of the OS-BFSAR system (including the GPS data of the vehicle-based radar) should be preprocessed. First, the GPS data should be transferred into the Cartesian coordinate system to obtain the local position of the vehicle-based radar. Second, the scattered data of the scene should be downsampled and filtered in the slow time. Finally, the scattered data must be range compressed by the matching filter function recorded before this experiment, and then the RFI in the scattered data should be suppressed by the method of the frequency spectrum equilibrium. In the imaging processing, the direct-path signal from the vehicle-based radar to the ground-based radar should be used to reduce or correct the time error of the scattered data caused by the uncertain time delay of the OS-BFSAR system. Figure 18 shows the imaging results of this measured data by the bistatic DTDA and proposed ETDA. In Figure 18, it is seen that the illuminated scene is well reconstructed by both two algorithms, and the metallic cylinders and metallic trihedral reflector are well focused, and the UWB features (i.e., the orthogonal and nonorthogonal sidelobes) can be clearly observed. Besides, it can be found that the image in Figure 18b obtained by the proposed ETDA is very close to that in Figure 18a obtained by the bistatic DTDA. However, the focusing quality of the image in Figure 18b is slightly degraded in comparison to that of the image in Figure 18a, since there may be still small phase error caused by the interpolations in the proposed ETDA. Fortunately, only the sidelobes of the focused image in Figure 18b may suffer from the effect of the phase errors.

In order to quantitatively evaluate the focusing quality of the SAR images in Figure 18, the resolutions of the focused trihedral reflector in the X and Y axes directions are measured based on the width of the amplitude profiles at −3 dB point. The measured resolutions in the X and Y axes direction obtained by the bistatic DTDA are 0.35 m and 1.73 m, respectively. And, the measured resolutions in the X and Y axes direction obtained by the proposed ETDA are 0.37 m and 1.87 m, respectively. Therefore, the measured resolutions by the two algorithms are very similar, which indirectly proves the validity of the proposed ETDA. Finally, for the sake of brevity, only the reconstruction time of the scene by the two algorithms is measured. The reconstruction time of the scene using the bistatic DTDA is 4687 s, while the scene reconstruction time using the proposed ETDA is 386 s. It can be concluded that the proposed ETDA is much faster than the bistatic DTDA while the achieved accuracies are about the same for the real OS-BFSAR imaging processing.

## 5. Conclusions

In this paper, an efficient time domain imaging method called ETDA is presented to give better performance in imaging efficiency than the bistatic DTDA for OS-BFSAR imaging processing. This method still inherits the advantages of the bistatic DTDA, such as the precisely accommodation of the large spatial variances, serious range-azimuth coupling and motion errors. Besides, it represents the subimages on polar grids in the ground plane instead of the slant-range plane, in order to be accurately referenced to the positions of both moving and stationary radars. Moreover, it derives the sampling requirements considering the motion errors for the polar grids to offer a near-optimum tradeoff between the imaging precision and efficiency. Experimental results based on both simulated and measured data confirm that the proposed algorithm performs better than the bistatic DTDA in terms of the efficiency improvement. The proposed ETDA for the BFSAR imaging processing with the complex configurations will be the next focus of our research.

## Figures and Tables

**Table 1:** Parameters of the simulated OS-BFSAR system.

Parameters	Values	Parameters	Values
Carrier frequency	700 MHz	Signal bandwidth	200 MHz
Sampling frequency	220 MHz	Pulse duration	1 μs
Pulse repetition frequency	120 Hz	Stationary radar position	(0,0,20) m
Moving radar ideal speed	45 m/s	Moving radar ideal altitude	100 m

**Table 2:** Measured parameters of the selected point-like scatterers.

Algorithms	Measured Parameters		ScattererC	ScattererE	ScattererG
Bistatic DTDA	Resolution (m)	Azimuth	0.9470	0.8863	0.8240
		Range	0.6702	0.6703	0.6705
	PSLR (dB)	Azimuth	−13.53	−13.60	−13.76
		Range	−13.22	−12.26	−13.26
	ISLR (dB)	Azimuth	−10.51	−10.41	−9.68
		Range	−10.21	−10.43	−9.73
Proposed ETDA	Resolution (m)	Azimuth	0.9486	0.8873	0.8288
		Range	0.6711	0.6713	0.6716
	PSLR (dB)	Azimuth	−13.47	−13.36	−13.87
		Range	−15.34	−15.19	−15.01
	ISLR (dB)	Azimuth	−10.62	−10.46	−9.71
		Range	−10.32	−10.49	−9.84

**Table 3:** Acquisition parameters of the real OS-BFSAR system.

Parameters	Values	Parameters	Values
Signal frequency	P-band	Sampling frequency	220 MHz
Pulse repetition frequency	500 Hz	Pulse duration	100 ns
Stationary radar position	(−54,0,6) m	Moving radar initial position	(−40,26,4) m
Moving radar ideal speed	12.8 km/h	Moving radar ideal altitude	4 m

## Abbreviations

The following abbreviations are used in this manuscript:

1PPS	One pulse per second
BP	Backprojection
SAR	Synthetic aperture radar
BSAR	Bistatic SAR
OS-BSAR	One-stationary BSAR
BFSAR	Bistatic forward-looking SAR
OS-BFSAR	One-stationary BFSAR
BLSAR	Side-looking SAR
OS-BSSAR	One-stationary BSSAR
PAMIR	Phased array multifunctional imaging radar
UWB	Ultrawideband
PSLR	Peak sidelobe ratio
ISLR	Integrated sidelobe ratio
RFI	Radio frequency interfere
FDA	Frequency-domain algorithm
TDA	Time-domain algorithm
DTDA	Direct TDA
ETDA	Efficient TDA
BPA	Backprojection algorithm
FBPA	Fast BPA
FFBPA	Fast factorized BPA
RDA	Doppler algorithm
OKA	Omega-k algorithm
CSA	Chirp scaling algorithm
NLCSA	Nonlinear CSA
P2P	Polar subimages into polar subimages
P2C	Polar subimages into the Cartesian image
FFT	Fast Fourier transform
IFFT	Inverse FFT
GPS	Global positioning system
VCO	Voltage-controlled oscillator
DPLL	Digital phase-locked loop
PC	Personal Computer
CPU	Central Processing Unit
GPU	Graphic Processing Unit
RAM	Random Access Memory
